# The relationship among psychopathology, religiosity, and nicotine dependence in Croatian war veterans with posttraumatic stress disorder

**DOI:** 10.3325/cmj.2018.59.165

**Published:** 2018-08

**Authors:** Marina Šagud, Božena Petrović, Maja Vilibić, Alma Mihaljević-Peleš, Bjanka Vuksan-Ćusa, Iva Radoš, Alen Greš, Vladimir Trkulja

**Affiliations:** 1 University of Zagreb School of Medicine, Zagreb, Croatia; 2 Department of Psychiatry, University Hospital Center Zagreb, Zagreb, Croatia; 3 Department of Psychiatry, University Hospital Center “Sisters of Mercy”, Zagreb, Croatia; 4 Department of Psychological Medicine, University Hospital Center Zagreb, Zagreb, Croatia; 5 Department of Pharmacology, University of Zagreb School of Medicine, Zagreb, Croatia

## Abstract

**Aim:**

To examine relationships among combat exposure, posttraumatic stress disorder (PTSD) symptoms, depression, suicidality, nicotine dependence, and religiosity in Croatian veterans.

**Methods:**

This cross-sectional study used Combat Exposure Scale (CES) to quantify the stressor severity, PTSD Checklist 5 (PCL) to quantify PTSD severity, Duke University Religion Index to quantify religiosity, Montgomery Asberg (MADRS) and Hamilton Depression (HAM-D) rating scales to measure depression/suicidality, and Fagerstrom Test for Nicotine Dependence to assess nicotine dependence. Zero-order correlations, cluster analysis, multivariate regression, and mediation models were used for data analysis.

**Results:**

Of 69 patients included, 71% met “high religiosity” criteria and 29% had moderate/high nicotine dependence. PTSD was severe (median PCL 71), depression was mild/moderate (median MADRS 19, HAM-D 14), while suicidality was mild. A subset of patients was identified with more severe PTSD/depression/suicidality and nicotine dependence (all *P* < 0.001). Two “chains” of direct and indirect independent associations were detected. Higher CES was associated with higher level of re-experiencing and, through re-experiencing, with higher negativity and hyperarousal. It also showed “downstream” division into two arms, one including a direct and indirect association with higher depression and lower probability of high religiosity, and the other including associations with higher suicidality and lower probability of high nicotine dependence.

**Conclusions:**

Psychopathology, religiosity, and nicotine dependence are intertwined in a complex way not detectable by simple direct associations. Heavy smoking might be a marker of severe PTSD psychopathology, while spirituality might be targeted in attempts of its alleviation.

Oxford Centre for Evidence-based Medicine level of evidence: 3

Posttraumatic stress disorder (PTSD) results from severe stressful experience(s) during traumatic events, such as war operations. According to the 5th edition of the Diagnostic and Statistical Manual of Mental Disorders (DSM-5), it is characterized by re-experiencing of stressful events (cluster B), avoidance of trauma-related stimuli (cluster C), negative thoughts or feelings (cluster D) and hyperarousal (cluster E) (1). Severity of the combat experience is largely thought to determine the severity of PTSD symptoms (2-6), but findings have been inconsistent (7). However, most studies did not account for co-existing depression, although nearly 50% of the war veterans with PTSD had it as the most common comorbid disorder in PTSD (8-10). While PTSD might be a risk factor for (subsequent) development of depression (10), it is unclear whether the conditions tend to amplify each other, ie, the more severe PTSD, the more severe depression (11), or not (9).

Two phenomena have been extensively investigated in relation to PTSD psychopathology: smoking, or nicotine dependence, and religiosity. Prevalence of smoking is higher in PTSD patients including Croatian (12,13) or other war veterans (14,15) than in the general population and more intense smoking is associated with more severe symptoms (15-18). The association between PTSD and smoking seems to be bidirectional. Patients might smoke in an attempt to self-medicate to reduce tension and negative affect and symptoms after exposure to a trauma-related context (19). Prospective studies in stress-exposed individuals suggested smoking as a risk factor for developing PTSD symptoms (20), as it might potentiate or maintain maladaptive responses (21). Religiosity might also play a role in coping with the traumatic stress, given that life-threatening situations may change beliefs about existential meaning (22), which is why war veterans might exhibit different religious assumptions and practices than the general population (23).

Religiosity is a complex construct that may have opposite effects on health depending on the pattern of spiritual coping (24,25). Higher religious moral beliefs were beneficial to war veterans (17), whereas weakened religious faith and negative coping were associated with higher use of mental health services, more severe psychopathology, and suicidality (22,24). However, most studies have evaluated only individual relationship between PTSD and depression, smoking or dependence, and religiosity, without accounting for other factors intertwined with PTSD psychopathology. We assumed that considering all these factors in a stepwise analytical process accounting for confounding and possible moderating or mediating effects would improve our understanding of the complex nature of their inter-relationships. Out aim was to explore the relationships between severity of the core PTSD symptoms (overall and by clusters), depression, suicidality, religious involvement, and smoking in a sample of Croatian Homeland war veterans with combat-related PTSD.

## PATIENTS AND METHODS

This exploratory cross-sectional study was conducted between November 2017 and May 2018 at the University Hospital Center Zagreb and University Hospital Vrapče, Zagreb, which are among the largest national centers providing care for the Croatian Homeland war veterans with PTSD. Although thousands have been treated since 1991, at the time of the study, approximately 350-400 was veterans with combat experience had been under regular psychiatric treatment or follow-up (monthly or temporarily hospitalized) for ≥2 years. Those treated by the authors (MŠ, AMP, BVĆ, MV) were the candidates for inclusion.

### Patients

The study included consecutive male patients who met the following criteria: verified PTSD diagnosed in line with the DSM-5; age ≤65 years; and signed informed consent. For the purpose of the study, all patients were re-evaluated using the Structured Clinical Interview for DSM-5 (26) to confirm the diagnosis and presence of the exclusion criteria, which included dementia, intellectual disability or any other severe cognitive impairment, lifetime history of bipolar disorder, schizophrenia, schizoaffective or delusional disorder, and alcohol or drug dependence in the previous six months. At the time of the study, all patients were attending supportive psychotherapy and received pharmacological treatments consisting mainly of selective serotonin or dual reuptake inhibitors and/or benzodiazepines and, sporadically, low-dose quetiapine, promazine or olanzapine at bedtime.

### Method

*PTSD severity*. The PTSD Checklist for DSM-5 (PCL-5) is a validated self-reporting instrument that quantifies symptom severity overall and by the four PTSD clusters consisting of re-experiencing stressful stimuli (PCL-B), avoidance of trauma-related stimuli (PCL-C), negative thoughts or feelings (PCL-D), and hyperarousal (PCL-E). Higher scores (minimum 17, maximum 85) indicate worse symptoms (27).

*Severity of stressful stimulus*. Combat Exposure Scale (CES) is a validated self-administered instrument where higher scores (minimum 0, maximum 41) indicate more severe past combat-related stressful stimuli (28).

*Depression and suicidality*. Montgomery-Asberg Depression Rating Scale (MADRS) (29) and the 17-item Hamilton Depression Rating Scale (HAM-D) (30) are validated clinician-administered tools that quantify depressive difficulties over the past week. MADRS consists of 10 items (item 10 pertaining to suicidal thoughts) and HAM-D consists of 17 items (item 3 pertaining to suicidal thoughts). Higher scores (0-60 by MADRS, 0-52 by HAM-D) indicate more severe depression.

*Religiosity*. The Duke University Religion Index (DUREL) is a validated self-administered instrument for quantification of basic religious or spiritual traits testing for “organizational” religious activity (ORA), “non-organizational” religious activity (NORA), and intrinsic religiosity (IR) (31,32). Higher scores (minimum 5, maximum 27) indicate more intense religiosity.

*Smoking or nicotine dependence*. The Fagerstrom Test for nicotine dependence (FTND) is a validated self-administered instrument that quantifies the mode and extent of cigarette smoking (33). It categorizes the level of nicotine dependence as low, low-moderate, moderate, or high.

*Outcome measures*. The outcome measures for PTSD included total PCL score, scores on each of its clusters, and total CES score. The outcome measures for depression included total MADRS and total HAM-D scores and separately MADRS score without item 10 and HAM-D without item, and intensity of suicidal thoughts. MADRS item 10 and HAM-D item 3 address the same characteristic, but use different wording and different scoring of the answers. Therefore, MADRS item 10 and HAM-D item 3 were summed-up to represent a single measure of suicidality. The outcome measures for religiosity consisted of total DUREL score and ORA, NORA, and IR subscores. Patients were dichotomized as those with high (ORA score ≥4, NORA score ≥4, IR score ≥10) and non-high overall religiosity (31,32). Finally, patients were classified as either current smokers or non-smokers, and nicotine dependence was classified as none or low and moderate or high.

### Statistical analysis

Considering the exploratory nature of the study, data were analyzed in several steps. First, zero-order correlations (non-parametric Kendall’s correlation) were assessed between different outcome measures and cluster analysis was performed to screen for potential associations. Then, independent associations were identified using multivariate analysis of variance and logistic regression. Finally, based on these analyses, hypotheses were generated about inter-relationships that were evaluated using multivariate mediator models. We used SAS 9.4 for Windows (SAS Inc., Cary, NC, USA) software licensed to the University of Zagreb School of Medicine for data analysis.

## RESULTS

Of 117 screened patients, 11 refused to participate and 37 suffered psychotic episodes, alcohol abuse, or bipolar disorder. Of 69 patients included in the study, 71% met the criterion of high religiosity, 46% were regular smokers, and 29% had a moderate or high nicotine dependence (Table 1). PTSD symptoms were severe, whereas overall depression severity was moderate. The intensity of suicidal thoughts was mild and 25% of patients had previous suicidal attempts.

**Table 1:** Characteristics of 69 patients with combat-related posttraumatic stress disorder (PTSD)

Characteristic	No. (%) of patients
Age (median, range; years)	55 (39-65)
Marital status	
single	4 (5.8)
married	58 (84.1)
widowed	2 (2.9)
divorced	5 (7.3)
Have children	57 (82.6)
Employment status	
unemployed	16 (23.2)
employed	25 (36.3)
retired	28 (40.6)
Education level (highest achieved)	
elementary	6 (8.7)
high school	54 (78.3)
higher education	9 (13.0)
PCL score (median, range)	
PCL-B (re-experiencing)	18 (5-25)
PCL-C (avoidance)	8 (2-10)
PCL-D (negativity)	23 (8-35)
PCL-E (hyperarousal)	21 (7-30)
CES score (median, range)	23 (9-32)
Depression (median, range)	
MADRS score	19 (3-36)
HAM-D score	14 (1-25)
Suicidality (median, range)	
MADRS without item 10	19 (3-35)
HAM-D without item 3	14 (1-24)
mean MADRS 10 + HAM-D 3^†^	0.64 (0-8)
Attempted suicide	17 (24.6)
Religiosity (median, range)	
DUREL index score	18 (0-27)
ORA DUREL subscale score	3 (0-6)
NORA DUREL subscale score	3 (0-6)
IR DUREL subscale score	11 (0-15)
DUREL high religiosity	49 (71.0)
Regular smoker	32 (46.4)
Nicotine dependence	
none	41 (59.4)
low or low-to-moderate	8 (11.6)
moderate	12 (17.4)
high	8 (11.6)
Cardiovascular comorbidity^‡^	31 (44.9)
Diabetes mellitus	11 (15.9)

*CES – Combat Exposure Scale; DUREL – Duke University Religion Index; ORA – organizational religious activity subscale, NORA – non-organizational religious activity subscale, IR – intrinsic religiosity subscale; HAM-D – Hamilton Depression Rating Scale; MADRS – Montgomery-Asberg Depression Rating Scale; PCL – PTSD Check List (clusters B-E).

†Both MADRS and HAM-D scales quantify intensity of suicidal thoughts based on one question with different scoring. Some patients scored “0” on one scale and “1” on the other. Therefore, the sum of scores on these two scales was used.

‡Treated hypertension, any form of coronary artery disease, chronic heart failure, arrhythmia, peripheral artery disease, or history of stroke or transitory ischemic attack.

### Unadjusted associations

Higher DUREL score was associated with higher subscale scores and weakly with higher PCL-B score (Table 2). The same weak association with PCL-B was observed for ORA and NORA scores, but not for IR score. Neither DUREL nor any of its subscales were associated with any other measure. Higher CES was associated with higher total PCL and higher PCL-B and D, but not with PCL-C and E scores (Table 2). It was also weakly associated with higher MADRS score and higher nicotine dependence, but not with HAM-D score. Higher total PCL was associated with higher scores on its subscales, MADRS, HAM-D, suicidality, and nicotine dependence scores. PCL-B showed the same pattern, but it was not associated with PCL-C, while PCL-C was not associated with any other measure. Higher PCL-D and PCL-E were associated with higher depression (MADRS, HAM-D), suicidality, and nicotine dependence, whereas higher MADRS and HAM-D scores were associated with higher suicidality (Table 2).

**Table 2:** Zero-order correlations (non-parametric) between measures of religiosity (DUREL, ORA, NORA, IR), combat exposure (CES), overall PTSD symptom severity (PCL) or severity of re-experiencing stressful events (PCL-B), avoidance of related stimuli (PCL-C), negativity (PCL-D), and hyperarousal (PCL-E), depression (MADRS, HAM-D), suicidality (sum of MADRS item 10 and HAM-D item 3 scores), and nicotine dependence (“nicotine” – the 5 levels of nicotine dependence were considered as an ordinal variable with values from 0 [none] to 4 [high])*†

	DUREL	ORA	NORA	IR	CES	PCL	PCL-B	PCL-C	PCL-D	PCL-E	MADRAS	HAM-D	Suicidality	Nicotine
DUREL	—	0.689	0.717	0.799	0.064	0.084	0.210	-0.050	0.115	-0.023	-0.049	-0.089	-0.024	-0.008
ORA	<0.001	—	0.582	0.527	0.004	0.038	0.163	-0.055	0.022	-0.041	-0.107	-0.076	-0.046	-0.095
NORA	<0.001	<0.001	—	0.503	0.061	0.147	0.256	-0.006	0.163	0.040	0.021	-0.045	0.078	-0.086
IR	<0.001	<0.001	<0.001	—	0.035	0.014	0.096	-0.109	0.074	-0.076	-0.103	-0.144	-0.073	0.057
CES	0.456	0.970	0.505	0.687	—	0.230	0.351	0.038	0.209	0.132	0.177	0.094	0.031	0.193
PCL	0.321	0.677	0.101	0.871	0.007	—	0.536	0.336	0.751	0.703	0.490	0.454	0.287	0.383
PCL-B	0.015	0.079	0.005	0.278	<0.001	<0.001	—	0.127	0.361	0.367	0.292	0.292	0.245	0.198
PCL-C	0.586	0.574	0.947	0.240	0.674	<0.001	0.167	—	0.271	0.222	0.085	0.093	-0.003	0.169
PCL-D	0.178	0.811	0.072	0.394	0.015	<0.001	<0.001	0.003	—	0.551	0.453	0.366	0.223	0.376
PCL-E	0.791	0.653	0.662	0.388	0.124	<0.001	<0.001	0.015	<0.001	—	0.501	0.480	0.293	0.408
MADRS	0.567	0.245	0.819	0.241	0.040	<0.001	0.001	0.351	<0.001	<0.001	—	0.692	0.575	0.129
HAM-D	0.302	0.314	0.620	0.100	0.276	<0.001	0.001	0.310	<0.001	<0.001	<0.001	—	0.477	0.109
Suicidality	0.798	0.654	0.435	0.451	0.740	0.002	0.010	0.976	0.018	0.002	<0.001	<0.001	—	-0.081
Nicotine	0.930	0.358	0.399	0.558	0.045	<0.001	0.040	0.096	<0.001	<0.001	0.179	0.256	0.442	—

*CES – Combat Exposure Scale, DUREL – Duke University Religion Index, ORA – organizational religious activity subscale, NORA – non-organizational religious activity subscale, IR – intrinsic religiosity subscale, HAM-D – Hamilton Depression Rating Scale, MADRS – Montgomery-Asberg Depression Rating Scale, PCL –Post-Traumatic Stress Disorder (PTSD) Check List (clusters B-E).

†Kendall’s correlation coefficients are given by row (open cells) and corresponding *P* values by column (shaded cells)*

Cluster analysis indicated Cluster 1 with 25 subjects and Cluster 2 with 44 subjects that were fully comparable regarding religiosity, but Cluster 2 subjects had considerably higher CES and all PCL scores, depression, suicidality, and nicotine dependence (Table 3).

**Table 3:** Summary of cluster analysis for two clusters identified on the basis of combat exposure score), severity of posttraumatic stress disorder symptoms, severity of depression, and nicotine dependence level considered as an ordinal variable*†

	Cluster 1 (n = 25)	Cluster 2 (n = 44)	Cluster 2 - 1 (95% CI)	*P‡*
**Variables**				
DUREL total score (overall religiosity)	18.0	19.0	0.0 (-3.0 to 3.0)	0.913
ORA	3.0	3.0	0.0 (-1.0 to 1.0)	0.948
NORA	3.0	4.0	0.0 (0.0 to 1.0)	0.282
IR	12.0	11.0	-1.0 (-3.0 to 1.0)	0.415
Level of religiosity based on total DUREL				
high	19 (76.0)	30 (68.2)	-7.8 (-28.0 to 15.4)	0.431
non-high	6 (24.0)	14 (31.8)		
CES	20.0	24.0	4.0 (2.0 to 7.0)	0.003
Severity of PTSD				
PCL total score (overall)	51.0	78.0	28.0 (22.0 to 33.0)	<0.001
PCL-B (re-experiencing)	15.0	20.5	6.0 (3.0 to 8.0)	<0.001
PCL-C (avoidance)	6.0	9.0	2.0 (0.0 to 4.0)	0.003
PCL-D (negativity)	14.0	27.0	12.0 (9.0 to 15.0)	<0.001
PCL-E (hyperarousal)	15.0	24.0	9.0 (7.0 to 11.0)	<0.001
Severity of depression				
MADRS total score	12.0	23.0	10.0 (7.0 to 13.0)	<0.001
HAM-D total score	9.0	17.0	7.0 (5.0 to 9.0)	<0.001
Suicidality				
MADRS score without item 10	12.0	22.0	9.0 (6.0 to 12)	<0.001
HAM-D score without item 3	9.0	16.0	6.0 (4.0 to 8.0)	<0.001
MARDS item 10+HAM-D item 3	0.0	1.0	0.0 (0.0 to 2.0)	0.002
Attempted suicide	5 (20.0)	12 (27.3)	7.3 (-16.6 to 26.8)	0.572
Nicotine dependence				
ordinal	0.0	1.0	0.0 (0.0 to 2.0)	0.002
categorical				
none or low	24 (96.0)	25 (56.8)	-39.2 (-54.9 to -19.8)	<0.001
moderate/high	1 (4.0)	19 (43.2)	18.2 (3.8 to 32.1)	

*CES – Combat Exposure Scale, DUREL – Duke University Religion Index, ORA – organizational religious activity subscale, NORA – non-organizational religious activity subscale, IR – intrinsic religiosity subscale, HAM-D – Hamilton Depression Rating Scale, MADRS – Montgomery-Asberg Depression Rating Scale, PCL – posttraumatic stress disorder (PTSD) Check List (clusters B-E).

†Depicted are median values for continuous variables or number with percentage of patients for nominal variables by cluster and difference between the two clusters.

‡Mann-Whitney test for median difference or proportion difference.

### Independent associations

In a multivariate analysis of variance (MANOVA), total PCL score, PLC subscores, MADRS, HAM-D (without suicidality), and suicidality scores were simultaneous dependent variables, while high religiosity (vs low), moderate or high nicotine dependence (vs none or low), CES, age, and cardiovascular comorbidity or DM were independent variables (Table 4). There was no clear association between high religiosity and PTSD or depression. Moderate or high nicotine dependence was associated with higher total PCL score and its cluster D (negativity) and cluster E (hyperarousal), but there was no apparent association with cluster B (re-experiencing) and C (avoidance) scores. Moderate or high nicotine dependence was also associated with higher HAM-D. Higher CES was associated with higher PCL-B and no other measure. When “nicotine dependence” was replaced by “current smoker”, there was no overall effect of smoking (not shown).

**Table 4:** Summary of multivariate analysis of independent associations of high religiosity, moderate or high nicotine dependence, and combat exposure with severity of posttraumatic stress-disorder symptoms (total PCL and cluster subscores), depression (MADRS and HAM-D without suicidality items), and suicidality (adjusted for age and cardiovascular comorbidity or diabetes mellitus)*

	Religiosity	Nicotine dependence	Combat exposure score	Age	Cardiovascular comorbidity or DM
**Variables**	**high vs non-high**	**moderate/ high vs none/low**	**by 1 point**	**by 1 year**	**yes vs no**
Overall effect^†^(*P*)	0.127	<0.001	0.010	0.334	0.547
Individual outcomes^‡^ (difference, 95% CI)					
Total PCL score	1.74 (-0.61, 9.52)	16.0 (8.00, 24.0)	0.43 (-0.22, 1.09)	-0.39 (-0.95, 0.16)	4.18 (-2.84, 11.2)
*P*	0.678	<0.001	0.190	0.160	0.238
PCL-B score	1.09 (-1.22, 3.39)	1.81 (-0.57, 4.18)	0.31 (0.11, 0.50)	-0.09 (-0.25, 0.08)	1.35 (-0.73, 3.43)
*P*	0.351	0.133	0.002	0.291	0.199
PCL-C score	-0.83 (-2.24, 0.58)	0.70 (-0.75, 2.16)	-0.01 (-0.13, 0.10)	-0.02 (-0.12, 0.08)	0.93 (-0.35, 2.20)
*P*	0.245	0.336	0.817	0.645	0.151
PCL-D score	2.36 (-1.27, 5.98)	7.28 (3.55, 11.0)	0.18 (-0.13, 0.48)	-0.09 (-0.35, 0.17)	0.47 (-2.80. 3.74)
*P*	0.199	<0.001	0.254	0.476	0.775
PCL-E score	-0.72 (-3.51, 2.07)	6.13 (3.26, 9.00)	-0.01 (-0.24, 0.22)	-0.17 (-0.37, 0.03)	1.48 (-1.04, 3.99)
*P*	0.607	<0.001	0.928	0.090	0.245
MADRS without item 10	-1.15 (-4.81, 2.50)	3.23 (-0.53,6.99)	0.17 (-0.14, 0.48)	-0.14 (-0.40, 0.12)	2.34 (-0.96, 5.63)
*P*	0.530	0.091	0.274	0.283	0.161
HAM-D without item 3	-1.91 (-4.59, 0.78)	3.07 (0.30, 5.83)	0.01 (-0.21, 0.24)	-0.05 (-0.24, 0.14)	1.32 (-1.09, 3.75)
*P*	0.162	0.030	0.890	0.620	0.279
Suicidal thoughts^§^	-0.17 (-1.16, 0.83)	-0.67 (-1.69, 0.36)	0.04 (-0.04, 0.12)	-0.02 (-0.09, 0.05)	0.40 (-0.49, 1.29)
*P*	0.735	0.197	0.340	0.497	0.378

*DM – diabetes mellitus, CI – confidence interval, HAM-D – Hamilton Depression Rating Scale (17 items), MADRS – Montgomery-Asberg Depression Rating Scale, PCL – Post-Traumatic Stress Disorder (PTSD) Check-List.

†Multivariate analysis of variance for total PCL score, PLC clusters, MADRS, HAM-D, and suicidal thoughts scores as simultaneous multiple dependent variables; nicotine dependence, religiosity, combat exposure scale score, age, and cardiovascular comorbidity or DM as independent variables. The *P* value refers to an overall effect of an independent variable on multiple dependent variables based on Pillai’s trace statistic.

‡By fitting general linear models separately to each individual outcome, with religiosity, nicotine dependence, combat exposure scale score, age and cardiovascular comorbidity or DM as independent variables.

§MADRS item 10 and HAM-D item 3 summed.

In a reverse analysis, religiosity and nicotine dependence were dependent variables in logistic regression. Independent variables included CES, PCL-B, PCL-D, and E summed, HAM-D, suicidality, age, cardiovascular comorbidity or DM, and religiosity in the analysis of nicotine dependence and *vice-versa*. Highly religious patients were less likely to have moderate or high nicotine dependence, whereas those with moderate or high nicotine dependence were less likely to be highly religious (Table 5). However, the estimates were imprecise due to limited sample size. Higher PCL-D+E was associated with higher odds of moderate or high nicotine dependence, while higher suicidality was associated with lower odds. Higher HAM-D score was associated with lower odds of high religiosity.

**Table 5:** Summary of logistic regression analyses of high religiosity (vs non-high) and moderate or high nicotine dependence (vs none or low) outcomes*

	Moderate/high nicotine dep		High religiosity	
Outcomes	OR (95% CI)	*P*	OR (95% CI)	*P*
High religiosity	0.25 (0.04-1.31)	0.102	—	—
Moderate/high nicotine dependence	—	—	0.36 (0.06-1.81)	0.212
Combat exposure score	1.12 (0.96-1.34)	0.159	0.95 (0.83-1.06)	0.365
PCL re-experiencing (cluster B)	0.99 (0.77-1.31)	0.964	1.09 (0.94-1.29)	0.254
PCL negativity + hyperarousal (cluster D+E)	1.24 (1.11-1.45)	<0.001	1.05 (0.98-1.15)	0.182
HAM-D without item 3	1.01 (0.80-1.26)	0.917	0.84 (0.71-0.98)	0.025
Suicidality (MADRS item 10 + HAM-D item 3)	0.47 (0.19-0.83)	0.005	0.95 (0.65-1.44)	0.775
Age (by 1 year)	0.93 (0.82-1.05)	0.250	0.92 (0.83-1.01)	0.091
Cardiovascular comorbidity or DM	1.74 (0.35-10.2)	0.503	1.14 (0.34-3.84)	0.829

*OR – odds ratio, CI – confidence interval, PCL – Post-Traumatic Stress Disorder Check List, HAM-D – Hamilton Depression Rating Scale, MADRS – Montgomery Asberg Depression Rating Scale, DM – diabetes mellitus.

**Figure 1:**
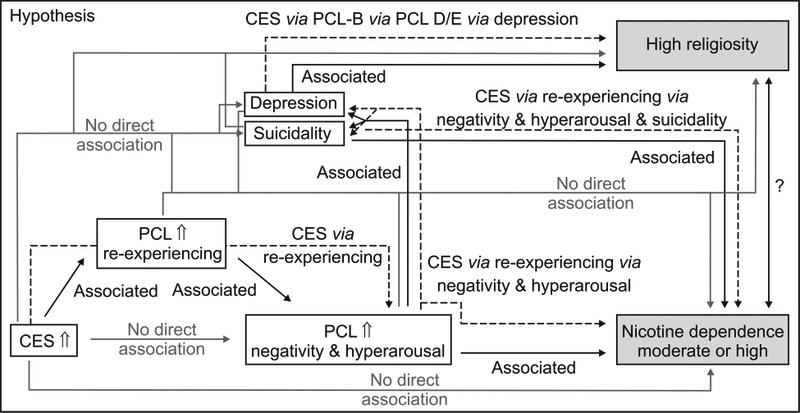
Schematic representation of sequential hypotheses about direct (full black lines/arrow) and a lack of direct (full gray lines/arrow) associations and about mediated (dashed black lines/arrows) associations between the intensity of stressful events (Combat Exposure Scale [CES] score,), intensity of posttraumatic stress disorder (PTSD) symptoms assessed by the PTSD Check List (PCL), in particular re-experiencing stressful events (PCL-B score) and negativity&hyperarousal (PCL-D and E scores), depression, suicidality, high religiosity and nicotine dependence. See text for detailed hypothesis about a “direct/mediated association chain”. The chain is generated based on univariate (unadjusted) associations depicted in Table 2 and Table 3 (zero-order correlations and cluster analysis) and independent (adjusted) associations depicted in Table 4 and Table 5 (multivariate analysis of variance and logistic regression).

**Figure 2:**
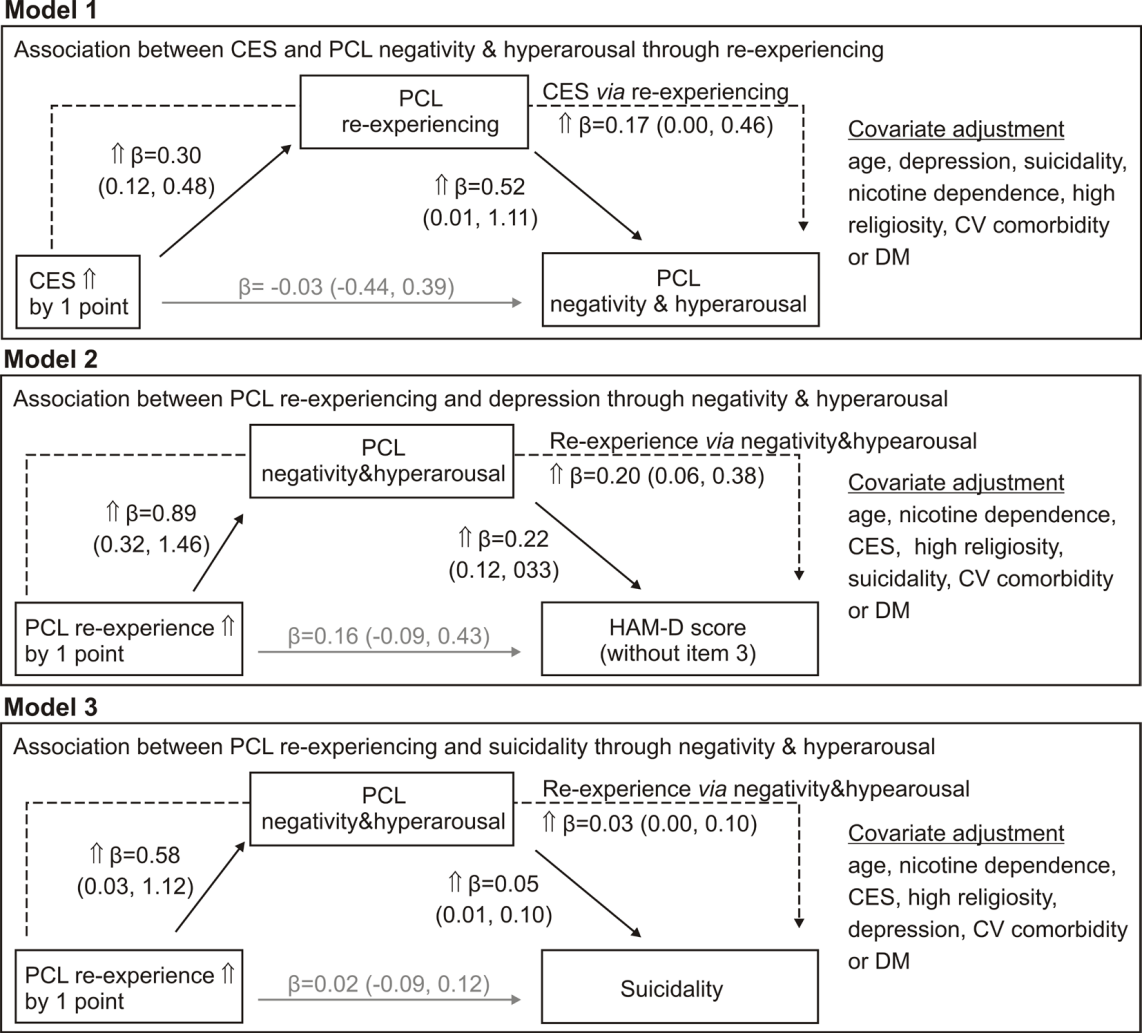
Schematic representation of mediation (based on multivariate regressions) models evaluating the hypotheses depicted in Figure 1: Models 1, 2 and 3. All models included the same variables as follows: combat exposure score (CES), posttraumatic stress disorder (PTSD) check list (PCL) cluster B score (re-experiencing), PCL cluster D+E score (summed scores of negativity and hyperarousal), Hamilton Depression Rating Scale (HAM-D) score without item 3 (depression without suicidality), suicidality score (summed scores on HAM-D item 3 and Montgomery-Asberg Depression Rating Scale item 10), religiosity level (high or non-high), nicotine dependence (moderate/high or none/low), age and presence of cardiovascular comorbidity (CV) or diabetes mellitus (DM). The roles of “independent”, “mediator” or “dependent” variable or covariates, changed across the models. All possible associations – direct or mediated – are adjusted for all variables in the models hence all are “adjusted” or independent. Associations are expressed as regression coefficients (β) with 95% confidence intervals. Covariate effects on mediator or dependent variables are omitted for clarity. Fonts and lines/arrows (full for direct associations, dashed for mediated associations) in black depict coefficients (associations) different from zero. Gray font/lines depict coefficients not different from zero.

**Figure 3:**
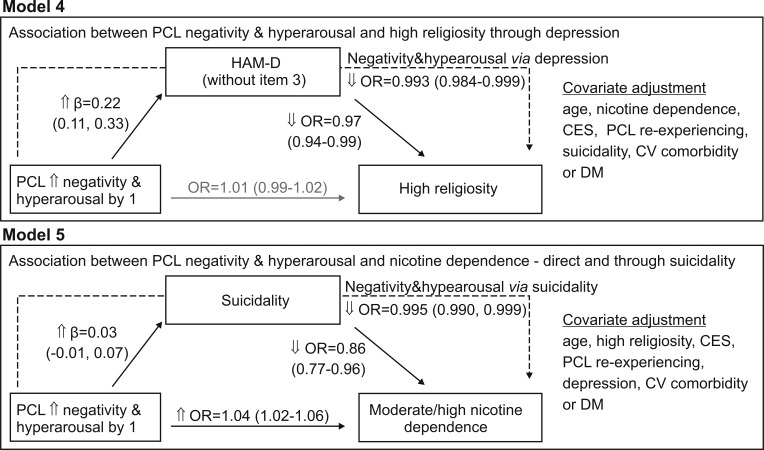
Schematic representation of mediation (based on multivariate regressions) models evaluating the hypotheses depicted in Figure 1: Models 4 and 5. All models included the same variables as follows: combat exposure score (CES), posttraumatic stress disorder (PTSD) check list (PCL) cluster B score (re-experiencing), PCL cluster D+E score (summed scores of negativity and hyperarousal), Hamilton Depression Rating Scale (HAM-D) score without item 3 (depression without suicidality), suicidality score (summed scores on HAM-D item 3 and Montgomery-Asberg Depression Rating Scale item 10), religiosity level (high or non-high), nicotine dependence (moderate/high or none/low), age and presence of cardiovascular comorbidity (CV) or diabetes mellitus (DM). The roles of “independent”, “mediator” or “dependent” variable or covariates, changed across the models. All possible associations – direct or mediated – are adjusted for all variables in the models hence all are “adjusted” or independent. Associations are expressed either as regression coefficients (β) or as odds ratios (OR) with 95% confidence intervals (CI). Covariate effects on mediator or dependent variables are omitted for clarity. Fonts and lines/arrows (full for direct associations, dashed for mediated associations) in black depict coefficients/ORs (associations) different from zero/unity. Gray font/lines depict coefficients/ORs not different from zero/unity.

Based on the above observations, we hypothesized about direct and mediated associations (Figure 1). CES was a starting point for all hypotheses as follows: a) CES has no direct associations with depression, suicidality, negativity & hyperarousal (PCL-D+E), religiosity or nicotine dependence; b) higher CES is associated with higher re-experiencing (PCL-B); c) higher PCL-B is directly associated with higher PCL-D+E, but not with depression or suicidality, religiosity or nicotine dependence; d) association between CES and PCL-D+E is mediated through PCL-B; e) PCL-D+E has no direct association with religiosity, but it is directly associated with (i) nicotine dependence, hence CES or re-experiencing is associated with nicotine dependence through PCL-D+E, and (ii) depression and suicidality; f) depression has no direct association with nicotine dependence, but it is directly associated with religiosity, hence a mediation chain is CES/re-experiencing – PCL-D+E – depression – religiosity; g) suicidality has no direct association with religiosity, but it is directly associated with nicotine dependence, hence there are two mediation chains including (i) CES/re-experiencing – PCL-D+E – nicotine dependence and (ii) CES/re-experiencing – PCL-D+E – suicidality – nicotine dependence. To test these hypotheses, 5 models were fitted with the same variables: CES, PCL-B, PCL-D+E, HAM-D without suicidality (depression), suicidality, religiosity (high or non-high), nicotine dependence (moderate-high or none-low), age and cardiovascular comorbidity or DM. Independent, mediator, and dependent variables changed across the models (Figures 2 and 3). All associations, either direct or mediated, in each model were independent, adjusted for all other variables. In Model 1 (Figure 2), CES was an independent, PCL-B was a mediator, and PCL-D+E was a dependent variable, and all other variables were covariates. Higher CES was directly associated with higher PCL-B, but not with PCL-D+E; higher PCL-B was directly associated with higher PCL-D+E, hence higher CES was associated with higher PCL-D+E through PCL-B. The starting, independent variable in Model 2 and Model 3 was “shifted down the chain”, from CES (now a covariate) to PCL-B. In both models, higher PCL-B was directly associated with higher PCL-D+E, but not with depression (Model 2) or suicidality (Model 3) (Figure 2). Higher PCL-D+E was directly associated with higher depression (Model 2) or higher suicidality (Model 3). Therefore, in both models, there was a mediated association between PCL-B and the dependents (Figure 2). In Models 4 and 5, PCL-D+E was the independent, depression (Model 4) or suicidality (Model 5) were mediators, and high religiosity (Model 4) or moderate/high nicotine dependence (Model 5) were dependent varables (Figure 3). In Model 4, higher PCL-D+E was directly associated with higher depression, but not with high religiosity, and higher depression was directly associated with lower odds of high religiosity; hence, higher PCL-D+E was associated with lower odds of high religiosity through depression. In Model 5, higher PCL-D+E was directly associated with higher suicidality and higher odds of moderate or high nicotine dependence. Higher suicidality was directly associated with lower odds of moderate or high nicotine dependence; hence, higher PCL-D+E was associated with lower odds of moderate or high nicotine dependence through suicidality. Higher PCL-D+E was associated with moderate or high nicotine dependence in opposing ways, ie, with higher odds directly, and with lower odds through suicidality (Figure 3).

## DISCUSSION

### Combat experience and PTSD

Our results showed that PTSD is a chronic disorder that does not subside with time (6,34). Higher combat exposure was associated with more intense re-experiencing, negativity and hyperarousal, and depression, supporting the view that it predicts PTSD persistence (35). A previous study in Croatian veterans found that all PTSD symptoms were more severe with more intense war experience, but it did not assess depression and negativity and participants were exclusively prisoners of war (2). Different stressors impact PTSD clusters differently, eg, re-experience is associated with violent combat, but it does not seem to be associated with avoidance symptoms (3). In the present study, avoidance was not related to any measure, which is in line with the observations that avoidance changes over time, while re-experiencing remains stable (3). Avoidance seems to be linked to combat severity only early in the PTSD course, primarily in younger individuals, and the relationship might include alcohol dependence (3,5). Our study participants were middle-aged veterans without recent alcohol abuse or dependence. One previous report found no direct link between combat exposure and suicidal thoughts (11). Our findings suggest that suicidality is linked to traumatic experience indirectly, through re-experiencing, negativity, hyperarousal, and depression.

### Nicotine dependence and psychopathology

We found a high smoking prevalence in our study participants, but the smoking status was not associated with PTSD severity, which is in line with previous findings (13). However, moderate or severe nicotine dependence was associated with more severe negativity, hyperarousal, and depression. This is also in agreement with several reports (15-17), particularly regarding hyperarousal symptoms (8,15,19).

We observed no association between nicotine dependence and core depression, but higher suicidality seemed to be associated with lower nicotine dependence. These two observations seem contradictory. Patients with more severe PTSD, depression, and suicidality also had a higher prevalence of moderate or high nicotine dependence, whereas higher suicidality scores were directly associated with lower odds of moderate or high nicotine dependence. However, cluster analysis does not control for confounding, whereas regression analyses detect independent associations. Also, the association between suicidality and nicotine dependence is not only a direct one. Our Model 5 showed that higher negativity and hyperarousal were directly associated with higher suicidality and moderate or high nicotine dependence and with lower odds of moderate or high nicotine dependence indirectly through their association with suicidality. Therefore, it is not surprising that higher negativity and hyperarousal, higher suicidality, and moderate or high nicotine dependence were clustered, but at a given level of negativity and hyperarousal, higher suicidality seemed to be associated with lower odds of moderate or high nicotine dependence. This finding seems counterintuitive given that tobacco use was repeatedly associated with suicidality (36-38). The question is whether smoking provokes suicidality or suicidal individuals smoke as a self-medication. In the present study, patients with higher disease severity, which itself is a stressor, had higher levels of nicotine dependence. Since the rewarding effects of nicotine are augmented after exposure to stressors, at least in animal models (39), nicotine might exert distinct effects in highly-stressed individuals with PTSD that decrease suicidality, but not other symptoms. Smoking cessation, in turn, might increase suicidality (36). None of our patients attempted to quit smoking at the time of the assessment.

### Religiosity and psychopathology

In the US war veterans, different aspects of higher religiosity were associated with milder PTSD symptoms and depression (40,41). We also found that more severe depression (higher HAM-D without item 3) was associated with lower odds of high religious involvement (based on DUREL), while more intense PTSD symptoms (re-experiencing, negativity, and hyperarousal) were also associated with lower odds of high religiosity, albeit not directly as the association seemed to be mediated through depression. Studies in war veterans with PTSD reported inverse relationships between general religiosity and suicidality (42,43). These studies included generally more severely suicidal patients. We intentionally separated depression and suicidality, because core depression and suicidality have different clinical meaning and suicidality in our study sample was generally mild and minimally contributed to the overall depression scores. Both were evaluated in models with mutual adjustments. Under such circumstances, we observed no association between religiosity and suicidality; however, depression and suicidality were closely correlated. Our results suggest that in moderately depressed but mildly suicidal combat PTSD patients, high religiosity was primarily associated with (lower) depression and association with suicidality existed to the extent to which it was a part of overall depression.

Our exploratory analysis of complex constructs had several limitations. The exploratory nature particularly refers to mediation analysis which *a priori* implies causal relationships, while the study was cross-sectional. Due to a limited sample size, most estimates were imprecise. Furthermore, the cultural setting of the present study was specific as it evaluated relationship between religious involvement and psychopathology in war veterans 20 years after the end of the war that marked the beginning of a post-communist era. On the other hand, participants were enrolled consecutively based on restrictive criteria to enable evaluation of targeted psychopathology, ie, core PTSD and depression, and religious involvement and smoking. In combination with statistical adjustments, this approach allowed for a fair control of confounding, and the present observations could be considered internally valid to a reasonable extent. Although the sample size was limited a consequence of timing of the study and applied inclusion and exclusion criteria, we interpreted data with caution focusing on size of the “effects” and their direction. Finally, using CES score, albeit assessed retrospectively with susceptibility to recall bias, as a starting point in the mediation chains followed by PTSD elements, seemed to be psychodynamically justified. Under these circumstances, we observed a rather straightforward association between higher religious involvement and lower depression severity. The coexisting direct and mediated relationships were indicated between the severity of combat trauma, clusters of core PTSD (re-experiencing, negativity and hyperarousal), depression, and religious involvement on one hand, and suicidality and nicotine dependence, on the other. While the present work cannot exclude other possible relationships, the apparent ones deserve further evaluation.

In conclusion, our results suggest complex associations between war stressors, psychopathology, nicotine dependence, and religiosity in patients with combat-related PTSD. Specifically, direct and mediated associations seem to exist between severity of the past stimuli and different aspects of current psychopathology. If one is to perceive religiosity as spirituality, data suggest that treatments targeting individual’s spiritual resources could directly or indirectly improve the management of PTSD, which is in line with the currently suggested modifications of the standard cognitive processing therapies (44).
